# From Food Additives to Neurodegeneration: The Emerging Role of Polyphosphates in Tauopathies

**DOI:** 10.1021/acschemneuro.5c00915

**Published:** 2026-05-05

**Authors:** Lorenzo Barolo, Maria Vittoria Farina, Giovanna Cimaglia, Ylenia Gigante, Anna Mirone, Lorenza Mautone, Alberto Boffi, Silvia Di Angelantonio, Paola Baiocco

**Affiliations:** † D-Tails srl BC, 00165 Rome, Italy; ‡ Center for Life Nano- & Neuro-Science@Sapienza, 121451 Istituto Italiano di Tecnologia, V.le Regina Elena 291, 00161 Rome, Italy; § Department of Biochemical Sciences “Alessandro Rossi Fanelli”, 9311 Sapienza University of Rome, P.le A. Moro 5, 00185 Rome, Italy; ∥ Department of Physiology and Pharmacology, Sapienza University of Rome, P.le A. Moro 5, 00185 Rome, Italy

**Keywords:** Tauopathies, Neurodegeneration, Tau fibrillation, Polyphosphates, Mitochondrial dysfunction, Aggregation cofactors, Processed Food, Food Industry

## Abstract

Neurodegenerative diseases are characterized by progressive molecular and biochemical dysfunctions that disrupt neuronal homeostasis, leading to impaired nervous system function. In tauopathies, a specific class of neurodegenerative disorders, tau protein aggregation and mitochondrial dysfunction are pathological processes interconnected in a self-reinforcing cycle. In fact, tau fibrils impair mitochondrial transport, bioenergetics, and quality control, while mitochondrial dysregulation causes tau post-translational modifications, detachment from neurons, and aggregation. In this context, inorganic polyphosphates located in cells are recently emerging as a possible modulator of both tau aggregation and mitochondrial dysfunction, thereby contributing to the onset and progression of tauopathies, including Alzheimer’s disease. Additionally, inorganic polyphosphates are widely present in diets worldwide as food additives, suggesting a possible frightening connection between nutrition and tauopathies, especially in vulnerable individuals. Understanding these biochemical and nutritional interactions may support the development of novel therapeutic approaches and provide effective preventive strategies to mitigate the risk of neurodegeneration in aging populations. This review explores the current state of the art for *in vivo* and *in vitro* studies, exploring the role of endogenous polyphosphates in tau aggregation and mitochondrial dysfunction, including a novel focus point: how exogenous polyphosphates present in everyday processed food could potentially facilitate the onset of pathological conditions in humans.

## Graphical abstract

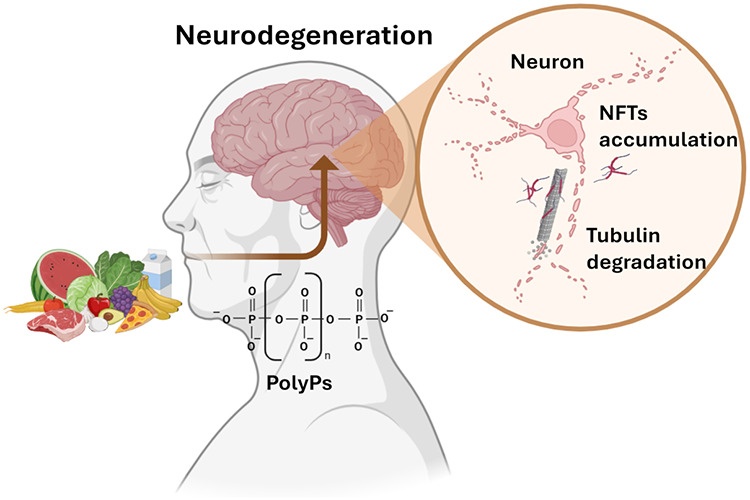

## 1 Introduction

### 1.1 Tauopathies

Neurodegeneration is a hallmark of several disorders, including Alzheimer’s disease (AD),^1^ Parkinson’s disease, and Huntington’s disease. It is characterized by progressive molecular and cellular alterations that lead to neuronal and motor dysfunction, culminating in a progressive decline in cognitive functions, memory loss, language deficiency, and movement impairment.^2^ Key processes of neurodegenerative disorders include a combination of multiple factors that disrupt normal cellular activities: (i) aberrant fibrillation and aggregation of endogenous proteins such as β-amyloid, tau, TDP-43, and α-synuclein;^3–6^ (ii) mitochondrial dysfunction;^7,8^ (iii) neuroinflammation;^9^ (iv) vascular dysregulation;^9^ and (v) failure of proteostasis.^10^ Among neurodegenerative diseases, tauopathies represent a distinct group of disorders in which aggregation of endogenous tau protein plays a central pathophysiological role. Tauopathies can be classified as primary or secondary, depending on whether tau pathology is the primary driver or occurs in association with other pathogenic factors, such as β-amyloid deposition in AD, or TDP-43 pathology in frontotemporal dementia (FTD) and amyotrophic lateral sclerosis (ALS).^6^ Additionally, mitochondrial dysfunction in tauopathies has gained interest over the years. Together with tau aggregation, they create a self-reinforcing vicious cycle of physiological disruption of cellular activity and directly contribute to neurodegeneration. In fact, aggregated tau species disrupt mitochondrial integrity and function, while mitochondrial stress promotes tau post-translational modifications (PTMs) and aggregation.

This review will specifically focus on the role of polyphosphates (polyPs) in tau protein fibrillation, exploring their potential connection to mitochondrial dysregulation. For example, it has been suggested that in tauopathies, endogenous polyPs are involved in the formation and accumulation of tau aggregates and in neuronal degeneration.^11–13^ Moreover, endogenous polyPs have a crucial role in mitochondrial dysfunction, influencing membrane potential, respiration, and susceptibility to stress, leading to oxidative stress and impaired ATP synthase activity in pathological conditions.^14–17^ Additionally, this review will shed light on the possible links between nutrition and tauopathies. In fact, polyPs are widely used as food additives and thus consumed in everyday diets worldwide.^18–21^ Exogenous polyPs are generally recognized as safe and healthy individuals can absorb them as free orthophosphate without complications.^22,23^ However, elderly people and pathological patients can exhibit impaired exogenous polyP intake and biodistribution, which could lead to possible accumulation of potentially harmful molecules.^24^ This review will analyze in-depth endogenous and exogenous polyP involvement in tauopathies.

### 1.2 Tau Fibrillation

Tau is a soluble disordered protein predominantly expressed in neurons.^25^ Under physiological conditions, tau binds microtubules to facilitate axonal structural integrity and stabilization, and to permit signal transport.^25^ It is encoded by the *MAPT* gene and in the adult human brain it exists in six isoforms due to alternative splicing of exons 2, 3, and 10.^25^ The tau protein sequence is generally organized into distinct domains: an N-terminal domain, a proline-rich region (PRD), a microtubule-binding region (MTBR) divided into 4 repeats (R1, R2, R3, and R4), and a C-terminal domain. The differences among isoforms are related to the number of repeats present in the MTBR and the N-terminal inserts.^25^ Structurally, the hexapeptide motifs at the beginning of the R2 and R3 repeats (PHF6* and PHF6, respectively) are considered the minimal essential elements for microtubule binding and aggregation. These motifs typically adopt a β-turn conformation in solution but a transition to an extended β-strand conformation was reported to be associated with a substantial effect on the kinetics of dimerization and fibril formation.^26^ Further structural studies of tau fibrils have demonstrated that the MTBR is responsible for critical contacts with C-terminal residues. In typical AD fibrils, the MTBR region spanning the PHF6* motif (specifically VQIINK) and PHF6 (VQIVYK) packs against C-terminal residues 373–378 (THKLTF).^4^ This interaction is believed to contribute significantly to the conformational stability and aggregation propensity of tau protein, thus increasing both tau fibril stability and polymorphism. Tau domains and fibrils are represented in Figure 1. The MTBR is notably rich in lysine residues, accounting for over 40% of the basic residues in the full-length protein (Figure 1A). It is also enriched in serine residues, which, under stress or pathological conditions, become susceptible to undesired PTMs, such as phosphorylation, acetylation, and truncation.^27^ Aberrant hyperphosphorylation leads to tau structural and behavioral modifications, lowering protein affinity with the microtubules and promoting tau detachment from the axons.^28^ Truncation and acetylation lead to enhanced aggregation and resistance to proteolysis.^29^ Additionally, each repeat within the MTBR terminates with a highly conserved PGGGX motif, where “X” is a positively charged residue in three out of the four repeats, suggesting a structural or regulatory role (Figure 1A).

**Figure 1:**
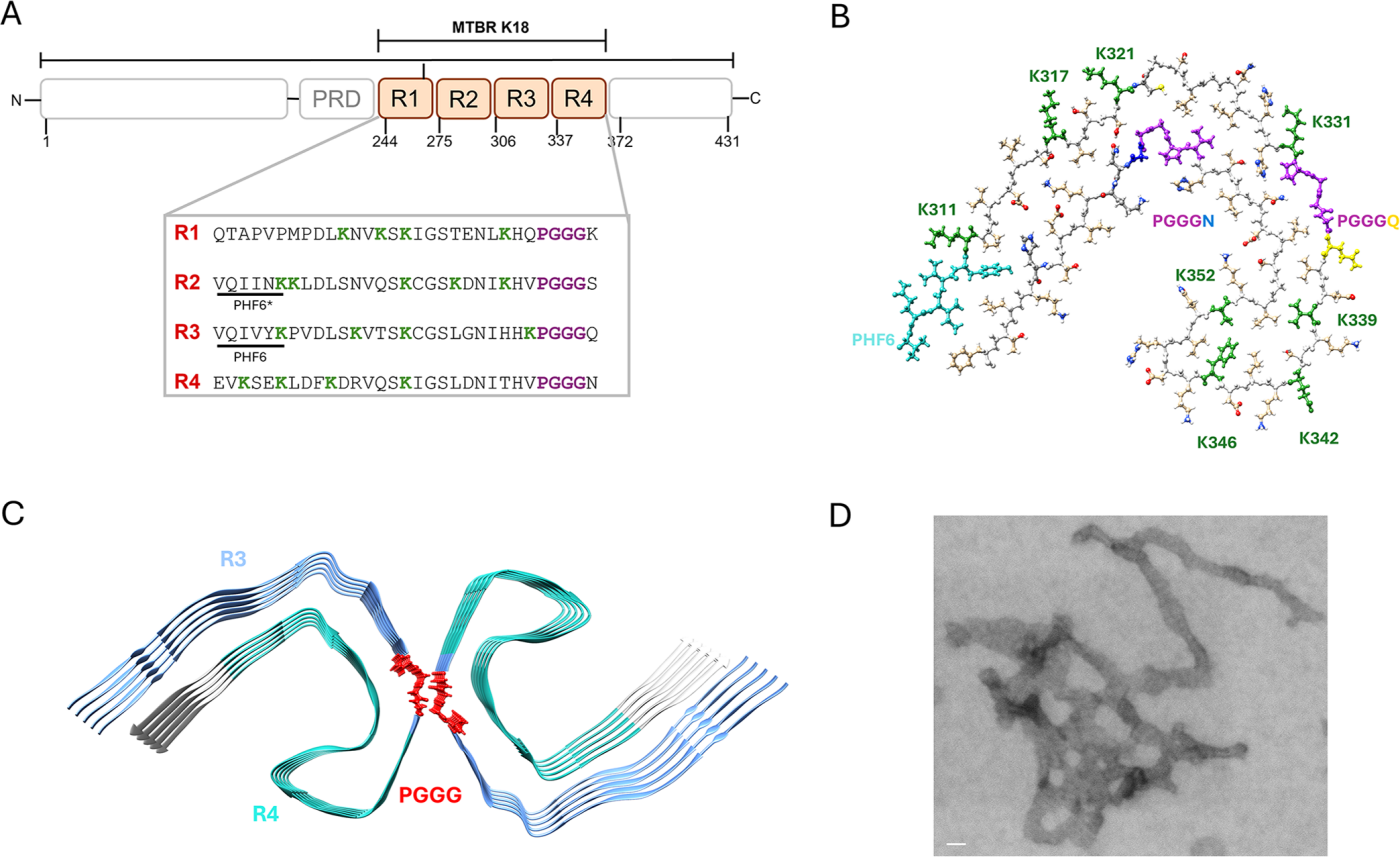
Structural and biochemical characterization of tau repeat domains and their role in filament formation. (A) Schematic representation of the full-length isoform (2N4R) of tau protein containing the MTBR domain, highlighting the four repeats (R1–R4). Below, the amino acid sequences of each repeat are shown with key functional motifs emphasized: the PHF6* (in R2) and PHF6 (in R3) motifs are underlined, conserved lysine residues (K) are shown in green, and the PGGG motifs are highlighted in purple; (B) atomic structure of the tau filament core (PDB: 5OL3), displaying side chains of residues critical for stabilizing the fold; (C) structural model showing the association of tau repeat domains into filaments, with the R3 and R4 highlighted in blue and turquoise and β-sheet conformation of the PGGG motif highlighted in red within the filament core; (D) Electron microscopy image of negatively stained tau filaments and tangles, demonstrating their fibrillar morphology (scale bar 20 nm).

The misfolded, detached protein then accumulates in the cytoplasm and can adopt aggregation-prone conformations, exposing hydrophobic hexapeptides within the MTBR^30^ (Figure 1B). When a misfolded tau monomer encounters another monomer, oligomerization can start. In fact, the R3 regions of both monomers can link and form a stable bond, specifically the 3 glycines of residues 332–336, the PGGGQ motif (Figure 1C). These glycines are known as the GGG patch. These newly formed conformations start to nucleate into β-sheet formations called oligomers^31^ (Figure 1C). Tau oligomers are soluble and highly toxic and can assemble into paired helical filaments (PHFs) or straight filaments (SFs), which subsequently can aggregate into insoluble neurofibrillary tangles (NFTs)^4^ (Figure 1D). The mechanisms driving tau nucleation and fibrillation are multifaceted and are not fully understood. Following PTMs and tau detachment, the fibrillation cascade can begin. In this context, physiological polyanions (RNA, polyPs) may enhance conformational flexibility and promote the exposure of aggregation-prone motifs, especially the PHF6 sequence. This process generates a pool of misfolded tau monomers capable of adopting nucleation-competent conformers. This early nucleation step is called seeding, in which pathological tau aggregates prompt more physiological tau monomers to detach and start aggregating.^32^ Liquid–liquid phase separation (LLPS) occurs after tau detaches from microtubules.^33^ In fact, tau protein can condense in a protein-rich dense state, increasing local protein concentration and altering the microenvironment, thereby lowering the kinetic barrier for β-sheet formation.^33^ Pathological tau LLPS can be driven by multiple causes, mostly electrostatic interactions.^33^ Importantly, LLPS represents a dynamic and initially reversible intermediate state that can transition into a more solid, aggregation-prone phase through a liquid-to-solid maturation process. Following nucleation, fibril elongation proceeds through the addition of monomeric tau to the growing fibril ends. Subsequent fragmentation of fibrils and secondary nucleation events amplify the aggregate formation. Tau oligomers and fibrillar seeds can also propagate through the brain in a prion-like manner, transferring among neurons via extracellular vesicles, endocytosis, and trans-synaptic pathways,^31,34^ as shown in Figure 2. Tau detachment from microtubules, aggregation, and seeding are catastrophic events, leading to altered cytoskeletal integrity, axonal transport, and synaptic plasticity, finally resulting in neuronal dysfunction and death^4,30,32^ (Figure 2). Beyond the devastating effects on neuronal viability and function, the propagation of tau aggregates also directly perturbs mitochondrial homeostasis.^35–37^ In fact, pathological tau impairs microtubule-based transport of mitochondria, leading to synaptic energy starvation and oxidative stress.^37^ In addition, NFTs can alter physiological mitochondrial bioenergetics and quality control, coupling tau aggregates to mitochondrial failure.^35–37^

**Figure 2:**
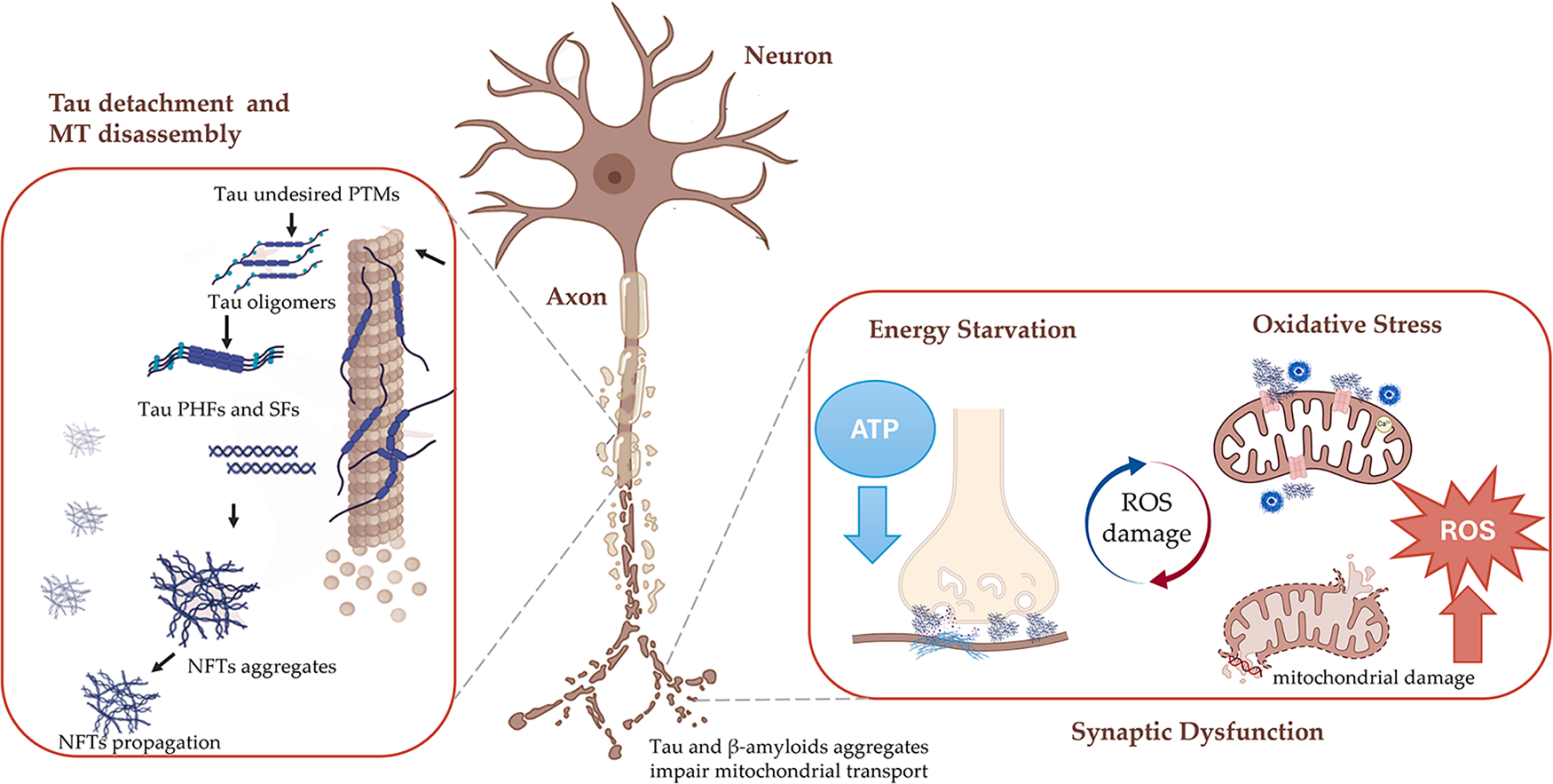
Schematic overview of neuronal pathological mechanisms in tauopathies. Two key pathological mechanisms among several neuronal alterations are illustrated: tau fibrillation cascade and mitochondrial dysfunction. At the axonal level, tau protein undergoes structural and functional modifications, leading to propagating aggregates that promote cytoskeletal disassembly and synaptic failure. Tau and β-amyloid aggregates accumulate at synapses, disrupting axonal trafficking and impairing mitochondrial transport. Reduced mitochondrial delivery lowers local ATP production, causing a synaptic energy deficiency. Tau-induced mitochondrial dysfunction compromises ROS regulation, promoting oxidative stress.

### 1.3 Mitochondrial Dysfunction

Numerous studies have recognized mitochondrial dysfunction as a hallmark of tauopathies.^8,38,39^ For example, elevated oxidative damage was observed in post-mortem brain tissue of AD patients,^8^ while mitochondrial disorders and mitophagy can be associated with FTD and ALS.^40,41^ Due to the high energetic demands of the brain, mitochondria play a vital role in supporting essential functions such as synaptic transmission and neuronal plasticity. Beyond ATP production, they are key regulators of calcium homeostasis, reactive oxygen species (ROS), and apoptosis.^42^ Impairments in mitochondrial function can exacerbate amyloid and tau pathology, promote oxidative stress, and amplify neuroinflammatory responses, thereby acting both as an early event and as a persistent driver of neurodegeneration.^8,43^ This dysfunction arises from a combination of complex, interconnected mechanisms that interact with and potentiate one another, contributing collectively to disease progression. These include impaired electron transport chain complex activity, which results in increased ROS production.^43^ Other contributing factors include genetic factors, such as the downregulation of ATP synthase β-subunit mRNA, mitochondrial DNA damage, and calcium dysregulation.^44,45^ In addition, pathological protein aggregates, such as β-amyloid and tau, strongly contribute to mitochondrial impairment. Specifically, β-amyloid peptides accumulate in the mitochondrial membrane, disrupting the function of the electron transport chain.^46^ This interference leads to impaired oxidative phosphorylation, ATP depletion, and increased levels of ROS, which damage mitochondrial DNA, proteins, and lipids (Figure 2). This reinforces oxidative stress and perpetuates a vicious cycle of cellular damage.^43,46^ More importantly, pathological tau is increasingly recognized as a fundamental driver and amplifier of mitochondrial dysfunction and dysregulation. In fact, tau fibrils impair mitochondrial morphology, transport, processes, permeability, and mitophagy. Pathological tau is correlated to defective axonal transport of mitochondria, depriving synapses of ATP and local Ca^2+^ buffering capacity. Moreover, phosphorylated tau can interact with regulators of mitochondrial fission and fusion, leading to degeneration of mitochondria and neurons.^36,37,47–49^ Beyond distribution and morphology, tau produces mitochondrial bioenergetic deficits, often termed mitochondrial energy starvation. It has been reported that pathological tau can alter the activity of complex I and V subunits. However, despite substantial functional evidence, the direct interaction between tau and complex I and V subunits remains to be fully elucidated. Furthermore, it is still unclear whether complex V inhibition represents a primary effect of tau toxicity or occurs as a result of upstream defects in complex I and overall respiratory chain dysfunction.^35,49^ However, it is evident that tau aggregates compromise the integrity and permeability of the mitochondrial membrane, thereby altering the conductance of the outer membrane,^36,49^ and directly interacting with mitochondrial transition pores and voltage-dependent anion channels, leading to loss of membrane potential, inhibition of ATP synthesis, and activation of apoptotic pathways.^36,50^ And finally, pathological tau can impair mitophagy, linking tau accumulation to defective removal of damaged mitochondria, further amplifying ROS.^51^ Together, these events initiate and sustain mitochondrial dysregulation, reinforcing the pathogenic cycle (Figure 2).

Given the importance of tau aggregation and mitochondrial dysfunction in tauopathies and the emerging data linking these two pathological processes to polyPs, this three-way interaction needs to be further elucidated. Considering that polyPs are daily consumed in diets worldwide and extensively used as food additives, this review analyzes exogenous polyPs as a potentially additional risk factor for tauopathies, establishing a nexus between biochemical mechanisms and nutritional exposures with the aim of promoting both therapeutic research and public health interventions.

## 2 Role of Polyphosphates in Tauopathies

### 2.1 Polyphosphates

PolyPs are linear molecules composed of inorganic phosphate units linked by phosphoanhydride bonds.^52^ They are conserved from bacteria to humans, and are involved in multiple cellular functions, i.e., energy metabolism, stress response, and regulation of protein activities.^52,53^ In humans, there are multiple types of polyPs, based on chain length.^54^ Short-chain polyPs are formed by 3 to 10 phosphate units, and are located in the cytoplasm, mitochondria, platelet dense granules, and lysosomes.^54,55^ The most famous short-chain polyP is adenosine triphosphate (ATP).^56^ Short-chain polyP functions are multiple, such as energy buffering, modulation of calcium signaling, clotting cascades, and more.^52,54^ Medium-chain polyPs present 10–60 units.^54^ They can be located in the mitochondrial matrix, the endoplasmic reticulum, and extracellular vesicles, and their role spans from modulation of protein folding to chelation of metal ions and mitochondrial permeability.^54,55^ Important medium-chain polyPs are platelet polyPs, involved in thrombin generation and coagulation.^57^ Long-chain polyPs go from 60 up to 1000 phosphate units.^54^ They are found in neutrophil extracellular traps (NETs) and chondrocytes.^54,58^ They are involved in inflammatory modulation and cytokine production, regulation of bone mineralization, and protein fibrillation under stress conditions.^54,55,58^ Long-chain polyPs are less present in humans compared to bacteria and yeasts. However, in humans, polyPs with 100–600 units are involved in lysosome activity.^59^ There is also another interesting category of polyPs, the phosphate-protein complex. Phosphorylation of proteins is one of the most important post-translational modifications in humans. It consists of the addition of a phosphate group to a specific amino acid residue, mostly serine (Ser), threonine (Thr), or tyrosine (Tyr). This reaction is catalyzed by kinases and reversed by phosphatases. Protein phosphorylation is a fundamental phenomenon with multiple applications in the human body. Adding or removing phosphate can switch on or off an enzyme, facilitate the formation or the disruption of protein complexes, determine subcellular localization, and prevent or facilitate degradation. Phosphorylation is especially important in neurons.^60^ In fact, this modification is involved in (i) synaptic plasticity, through the regulation of receptors such as AMPA, NMDA;^61,62^ (ii) in long-term potentiation, via CaMKII, PKA, PKC, and downstream kinases;^63,64^ (iii) in neurotransmitter release, by modulation of SNARE proteins and vesicle priming;^65,66^ and (iv) in tau regulation and interaction with microtubules, through hyperphosphorylation of the protein.^28,67^ The involvement of polyPs with tau fibrillation and mitochondrial dysregulation has started to shed light on the role of these molecules in neurodegenerative disorders like AD.

### 2.2 Polyphosphate Involvement in Tau Aggregation

Recent studies suggest that polyPs can modulate protein solubility and favor their stabilization into amyloid-like fibrils. They indicate that polyPs promote amyloidogenic fibrillar aggregation in a wide range of proteins, including globular proteins as well as intrinsically disordered proteins such as α-synuclein and tau.^68–70^ Focusing on tauopathies, polyPs can promote tau fibril formation, specifically under cellular stress conditions, especially oxidative. They can act as both initiators and stabilizers of tau fibrillation.^12,13,71,72^ The numerous negatively charged groups of polyPs, regardless of chain length, can interact with the positively charged or polar residues of tau, i.e., lysine (Lys), arginine (Arg), and glutamine (Gln), promoting structural reorganization and leading to nucleation and propagation of fibrils.^12,13^ PolyPs may induce aggregation due to three mechanisms: (i) direct binding, (ii) kinetic modulation, and (iii) indirect promotion.^12,13,73,74^ Direct electrostatic binding happens when negative phosphate groups interact with the positively charged domains of tau, specifically in its MTBR (R1–R4). This electrostatic bond promotes tau unfolding, exposing hydrophobic residues and stabilizing fibrillation-prone conformations, as represented in Figure 3. The kinetic modulation refers to the ability of polyPs to lower the energy barrier for tau nucleation, accelerating fibril formation.^12,13,71^ This property is dependent on chain length, with long-chain polyPs promoting aggregation more rapidly.^13^ Indirect promotion is linked to the ability of polyPs to modulate kinase activity.^74,75^ Specific kinases, such as GSK3β, CDK5, and MAPKs, are responsible for the pathological tau hyperphosphorylation.^74^ PolyPs may influence the activity of tau-relevant kinases/phosphatases, indirectly modulating the hyperphosphorylation of tau, and thus the accumulation of tau fibrils.^75^ Through direct or indirect promotion, polyPs may facilitate conditions for the formation and accumulation of the hyperphosphorylated prone-to-fibrillation protein.^12,13,73,74^ Oligomeric tau can then spread in the brain in a prion-like manner, initiating the fibrillation cascade in AD.^73^ Beyond their role in tau aggregation, recent findings indicate an additional disease-relevant function for polyPs in mouse models and patient-derived iPSC astrocytes carrying genetic mutations associated with other tauopathies, such as ALS and FTD.^11^ Dysregulated intracellular polyP levels in ALS/FTD models have been shown to contribute to motor neuron toxicity *in vitro*, identifying polyP concentration as a critical determinant of noncell-autonomous neurotoxicity and suggesting a potential link to tau-driven pathogenic processes across multiple neurodegenerative conditions.^11^

**Figure 3:**
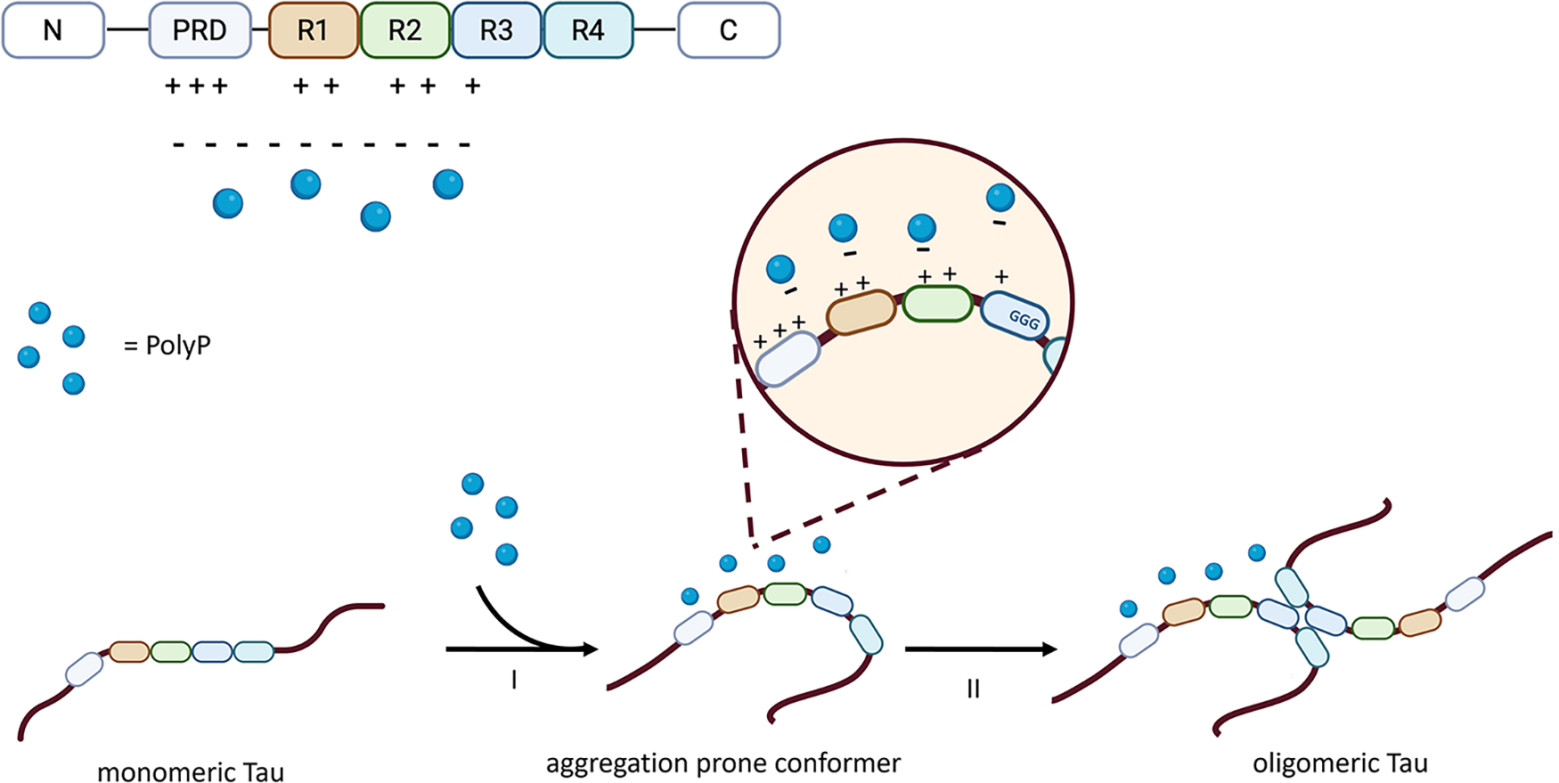
Polyphosphate-driven modulation of tau aggregation. Schematic representation of the interaction between polyPs and full-length tau. The domain organization of tau is shown at the top, including the N-terminal region (N), the proline-rich domain (PRD), the four microtubule-binding repeats (R1–R4), and the C-terminal region (C). PolyPs (blue spheres) interact electrostatically with positively charged residues enriched in the PRD and repeat domains. (I) PolyP binding induces conformational rearrangements in monomeric tau, promoting the exposure of aggregation-prone motifs (e.g., GGG patches; inset) and generating an aggregation-prone conformer. (II) This conformational transition facilitates intermolecular interactions, leading to tau oligomerization and subsequent fibril elongation.

### 2.3 Polyphosphate Involvement in Mitochondrial Dysfunction

As stated above, hyperphosphorylated tau and oxidative stress are also strongly linked to mitochondrial dysfunction, thus creating a pathologically vicious cycle including polyPs, tau fibrils, and impaired mitochondria.^36^ In fact, polyPs can interact with mitochondria indirectly, through tau, and directly. Several recent studies suggest that polyPs may be involved in mitochondrial dysfunction during tauopathies.^14,17^ In physiological conditions, polyPs support numerous fundamental mitochondrial processes, such as calcium homeostasis, ROS production, and apoptosis.^16,17^ As strong divalent-cation chelators, particularly of Ca^2+^, they modulate mitochondrial calcium uptake and storage, thereby influencing membrane potential and energy metabolism.^76^ However, in pathological conditions, polyPs can accumulate around mitochondria, leading to calcium overload in the matrix and opening of the mitochondrial permeability transition pore (mPTP).^77^ This event results in a loss of membrane potential, cytochrome c release, and apoptosis. PolyPs normally act as antioxidants and help regulate mitochondrial ROS by modulating key metabolic enzymes.^17,78^ During pathological circumstances, however, they can alter the activity of respiratory complexes, particularly complex I and III, promoting electron leakage and superoxide formation.^14,15^ This shift drives oxidative damage to mitochondrial proteins, lipids, and mtDNA, leading to impaired respiratory chain function and reinforcing a vicious cycle of ROS accumulation and mitochondrial dysfunction.^14–17^ Moreover, pathological polyP dysregulation can interfere with mitochondrial quality control pathways, including mitophagy and mitogenesis, limiting both the generation of new functional mitochondria and the clearance of the old dysfunctional ones.^14,17,79^ Additionally, ROS increase and oxidative stress caused by the interaction between polyPs and mitochondria can lead to tau phosphorylation and NFTs accumulation, further alimenting the pathological cycle.^80^

### 2.4 ATP Involvement in Tauopathies

An additional aspect worth exploring is ATP involvement in key processes of tauopathies, namely mitochondrial dysfunction and tau protein fibrillation.^81^ In pathological conditions, mitochondrial homeostasis is disrupted and ATP synthesis becomes a key point of vulnerability. For example, in AD, ATP synthase activity is impaired due to downregulation of the enzyme α-chain and aberrant modifications of its β-subunit.^82–84^ These alterations lead to reduced ATP production and extensive oxidative stress.^85^ In neurons, ATP is essential for ion gradients, neurotransmitter release, and synaptic plasticity.^86^ Therefore, an ATP deficiency can have profound effects on neuronal health. Additionally, ATP may also be involved in tau protein fibrillation.^81^ As a triphosphate, ATP can bind tau and promote its nucleation *in vitro*,^87^ although this process has not yet been analyzed *in vivo*. Moreover, in FTD/ALS, ATP modulates LLPS of TDP-43, leading to protein aggregation.^88^ Notably, impairments in ATP synthesis not only contribute to early pathological processes but also represent promising pharmacological targets for potential therapeutic intervention. Together, the interactions among polyPs, mitochondrial stress, ATP dysregulation, and inflammatory signaling converge to drive widespread energy failure, synaptic dysfunction, and ultimately neuronal death.

## 3 Analysis of Tau Fibrillation *In Vitro*

A deep understanding of the tau fibrillation mechanism is crucial to unraveling the molecular basis of tauopathies. Therefore, over the years, scientists have studied tau aggregation *in vitro* using different polyanionic cofactors to mimic physiological conditions and gain important information on the molecular mechanisms behind the onset of AD and other tauopathies.^12,13,87,89–92^ Frequently used anionic cofactors are heparin,^89^ RNA,^92^ arachidonic acid,^91^ synthetic compounds,^93^ and more recently polyPs.^12,13^ The cofactor helps to overcome the high solubility and structural plasticity of tau in its native form, enhancing the formation of fibrils. Apart from the fibrillation technique, the rest of the procedure is mostly conserved among the different studies. Fibrillation is typically induced by incubating tau constructs (20–100 μM) at 37 °C in PBS at pH 7.4, in the presence of a reducing agent and an anionic cofactor at various molar ratios. The obtained fibrils are analyzed using (i) multiple biophysical techniques, such as fluorescent probes, gel electrophoresis, immunoblotting, scanning electron microscopy, transmission electron microscopy, cryo-electron microscopy, and (ii) various biological systems, such as primary neurons, iPSC-derived human cells/organoids, and transgenic rodent models.^12,13,87,91,92,94–99^ However, different polyanionic cofactors will interact with tau protein in different ways.^12,93,95,101^ Therefore, the obtained fibrils will be different based on the cofactor, often drifting apart from the physiological aggregation condition.^4,12,94^ A summary table is provided here (Table 1).

**Table 1:** Comparative Analysis of Cofactors Involved in Tau Protein Aggregation Based on the Molecular Mechanism and Physiological Relevance

Cofactor	Structure	Mechanism of Action	Physiological Relevance	Advantages	Limitations
Heparin	Sulfated glycosaminoglycan	Electrostatic interaction; exposure of aggregation-prone R2-R3 tau regions	Low; not involved in tau fibrillation *in vivo*	Standard *in vitro* model, strong inducer	Polymorphic compound, nonphysiological fibrils, low reproducibility
RNA	Nucleic acid	Electrostatic interaction; LLPS	High; physiologically implicated in tau aggregation	Resembles physiological aggregation	Variability depending on RNA type and chain length
Arachidonic acid	Polyunsaturated fatty acid	Hydrophobic interactions via micelle formation	Medium; present in brain membranes, released under oxidative stress	Links to oxidative stress, AD relevance	Mechanism distinct from major cofactors, less studied
Dextran sulfate	Synthetic polyanion	Electrostatic interaction; mimics heparin	Low; synthetic compound	Avoids heterogeneity of heparin	Synthetic compound, nonphysiological fibrils
Polyphosphates	Inorganic polymers of phosphates	Electrostatic interaction, chain-length-dependent; stabilizes aggregation-prone conformations	High; present in the brain extracellular space, involved in the onset of tauopathies	Strong AD relevance, versatile mechanisms	Mechanism still unclear, chain-length-dependent

### 3.1 Tau Fibrillation *In Vitro* Using Heparin

Heparin is a heterogenic sulfated glycosaminoglycan and is considered the traditional standard polyanion used to induce fibrillation for all six isoforms of tau.^89,100^ It utilizes its strong negative charge to mimic the hyperphosphorylation that forces tau to detach from the microtubules and fibrillate.^89,100,101^ The aggregation mechanism is not clear at a molecular level; however, it is based on electrostatic interactions. In fact, after tau exposure to heparin, a bond between the negatively charged groups of heparin and the positively charged residues of tau is formed.^89,94^ This interaction promotes a conformational rearrangement of the protein, exposing aggregation-prone peptides in R2 and R3 regions, leading to tau monomers nucleation and subsequently to fibril elongation.^89,100–102^ Although heparin is considered the traditional standard for tau fibrillation *in vitro*, mainly due to its easy production and availability, this procedure presents several issues. Heparin salts produced and used *in vitro* present polymorphism and inherent propensity to aggregate, lowering the procedure effect and reproducibility.^89,94,101^ Moreover, human heparin does not seem to be involved in tau fibril formation or transportation during pathology onset, and heparin-induced fibrils do not resemble morphological features of those observed in the brain tissue of AD patients, strongly limiting the biological relevance of this analysis *in vitro*.^4,94^

### 3.2 Tau Fibrillation *In Vitro* Using RNA

Multiple forms of RNA, namely RNA transfer (tRNA) and polyadenylic acid (poly-A), have been used *in vitro* to promote tau fibrillation. It has been reported that chain length matters for fibrillation induction, specifically longer chains enhance aggregation.^92,103^ Similarly to heparin, RNA facilitates fibrillation by electrostatic interactions and LLPS.^92,102–104^ However, RNA-induced fibrillation *in vitro* resembles more physiological conditions. In fact, RNA is extensively present in humans, and its negatively charged groups are phosphates. Moreover, RNA plays an important role *in vivo* under physiological conditions during the onset of AD, enhancing the relevance of using RNA *in vitro*.^92,103^ However, RNA presents multiple disadvantages for *in vitro* applications. The RNA-tau fibrillation process is strongly dependent on procedure variables, such as stoichiometry and ion strength. Moreover, different RNA structures can act as different cofactors, complicating mechanistic interpretation and diminishing the *in vitro* relevance of the obtained fibrils.^92,105–107^

### 3.3 Tau Fibrillation *In Vitro* Using Arachidonic Acid

Arachidonic acid is a natural polyunsaturated fatty acid mostly found in eukaryotic cell membranes, especially in brain phospholipids.^91,108^ Arachidonic acid has been detected in the human brain, and interestingly, it is released from membranes in response to oxidative stress.^91,108^ Arachidonic acid promotes tau fibrillation *in vitro* through hydrophobic interactions, through micelle formation.^91,108^ The cofactor denatures the protein and exposes tau hydrophobic residues, forcing nucleation and subsequent fibrillation.^91,108^ Although its mechanism is different from all other polyanionic cofactors, arachidonic acid could still have a relevance for exploring tauopathy process, given the role of arachidonic acid in the human brain during oxidative stress, a condition often associated with the diseases.^91,108^

### 3.4 Tau Fibrillation *In Vitro* Using Synthetic Cofactors

Synthetic polyanions are artificial polymers with a high presence of negatively charged groups such as carboxylates, sulfonates, or phosphates.^93,105^ The most commonly used is dextran sulfate, a synthetic cofactor that mimics heparin, avoiding the heterogeneity of the natural compound.^93,105^ The aggregation mechanism is similar to those of other biological polyanions. However, different synthetic cofactors produce different fibrils, varying in length, width, and stability.^93,105^ Moreover, all tau fibrils obtained with synthetic polyanions do not resemble physiological filaments, lowering the biological relevance of this type of cofactor.^93,105^

### 3.5 Tau Fibrillation *In Vitro* Using PolyPs

As stated before, polyPs have recently gained importance for the production and analysis of tau fibrils.^12,13,52,71,109^ *In vivo*, short- and long-chained polyPs are present in the extracellular space of the brain, and are involved in many processes, such as cellular energy homeostasis, inflammation, ion channel function, and cell signaling in the mammalian brain.^16,54,55,76,110,111^ More importantly, polyPs compete with tubulin to bind tau, promote tau protein fibrillation into amyloid fibrils, and appear to be involved in the development and progress of tauopathies, for both tau aggregation and mitochondrial dysfunction.^11–14,71,109^ Although the exact mechanism of tau fibrillation with polyPs *in vitro* is still not fully elucidated, studies involving amyloidogenic proteins indicate that linear polyPs encourage the recruitment of monomeric and oligomeric structures into wrapped and elongated fibrils, thus preventing extensive gemmation and accumulation of oligomers and protofibrils.^12,13,109,110^ Compared with heparin-induced fibrils, these species have different chemical-physical properties and morphology and do not disassemble into oligomers or protofibrils.^12,94^ Interestingly, the efficacy of tau fibrillation appears to be related to polyP chain length, with longer polyP chains (>60Pi) showing greater effectiveness compared to shorter-chain polyP (<14Pi), probably due to increased charge and interaction surface.^13^ However, a recent paper showed how a short-chained polyP, specifically a tripolyphosphate, interacts with tau to form fibrils in a specific way.^12^ It was reported that the small polyP interacts with specific positively charged residues of the protein, stabilizing the aggregation-prone conformation and exposing its GGG patch that subsequently binds the other three glycines of another tau monomer, starting nucleation.^12^ In that case, the size of the polyP was fundamental to be encapsulated into the aggregation-prone conformation and therefore start fibrillation.^12^ This is very important given that ATP is chemically analogous to tripolyphosphates, and that its role in the onset of tau fibrillation and AD has been reported *in vivo*.^83,85,87,112^ However, the direct interaction of ATP with tau and its fibrillation mechanism have not been analyzed yet. Therefore, the role of short-chain polyPs in the onset of tauopathies becomes even more important.

## 4 Analysis of Mitochondrial Dysfunction *In Vitro*

*In vitro* analyses of mitochondria under pathological conditions became increasingly relevant to shed light on the possible mechanisms of dysregulation and the components involved. A plethora of protocols have been developed to assess the physiological or pathological state of mitochondria, based on monitoring specific hallmarks of mitochondrial dysregulation, such as (i) impaired oxidative phosphorylation,^42,43,113–115^ (ii) mtDNA mutations due to oxidative stress,^42,43,114,116,117^ (iii) interaction with β-amyloids, TPD-43, and tau aggregates,^36,40,41,50,118–122^ and (iv) reduced mitochondria biogenesis and defective mitophagy.^7,46,123–125^ Interestingly, *in vitro* analyses highlighted the pathologically vicious cycle linking mitochondrial dysfunction to polyPs and tau fibrils.^126,127^ Oxidative stress leads to tau hyperphosphorylation and NFTs, tau fibrils interact with mitochondria, causing organelle impairment, and mitochondrial dysfunction causes oxidative stress.^80^ Therefore, the information obtained on the role of polyPs and tau aggregates in mitochondrial dysfunction is fundamental to understanding the physiological behavior of the organelle and to better comprehend its early changes during the onset of tauopathies. Mitochondrial membrane potential (ΔΨ*m*) analysis studies the proton gradient essential for ATP production, which is altered by tau fibrils and polyPs during pathological conditions.^36,50,84,85,118–121,128–130^ Oxygen consumption rate (OCR) is a measure of oxidative phosphorylation, which is reduced by tau aggregation and polyP depletion.^9,130–133^ ATP quantification is obviously a very important analysis, given the fundamental role of this compound for synaptic and metabolic activity.^84,85,87,88^ Mitochondrial morphology and dynamics are also very important parameters to analyze. In fact, during tauopathies, mitochondria show increased fragmentation, often induced by interaction with tau aggregates.^46,50,123,125,126,134^ Mitochondrial biogenesis and mitophagy are also altered in pathological conditions, with the former reduced and the latter impaired, leading to the accumulation of damaged mitochondria.^46,50,123–126,134^ Calcium homeostasis measures the regulation of Ca^2+^ by the organelle, which is impaired by polyPs and tau during AD and other tauopathies, leading to cell death.^42,77,128,135,136^ Membrane permeability is another important parameter to study. Both polyPs and tau aggregates trigger opening of the transition pores of the membrane, leading to mitochondrial rupture and apoptosis.^46,130,137^

## 5 Polyphosphates in Nutraceutics and the Food Industry

PolyPs are regular components of diets worldwide and authorized as food additives for multiple critical functions including emulsification, moisture retention, and stabilization.^18–21^ When ingested, inorganic polyphosphates added to foods are largely degraded in the gastrointestinal tract,^138^ where enzymes involved in polyP synthesis and hydrolysis, such as polyphosphate kinases and exopolyphosphatases, are highly conserved across species. Approximately 80–90% of dietary polyPs are absorbed in the form of free orthophosphate, while phosphate in excess is excreted predominantly via the kidneys through glomerular filtration and tubular processing.^22,23^ In Western diets, total phosphate intake typically ranges from 1 to 2 g per day, although actual exposure may be considerably higher in individuals consuming large amounts of processed foods.^139,140^ A schematic overview of the principal applications of polyPs in the food industry is presented in Figure 4.

**Figure 4:**
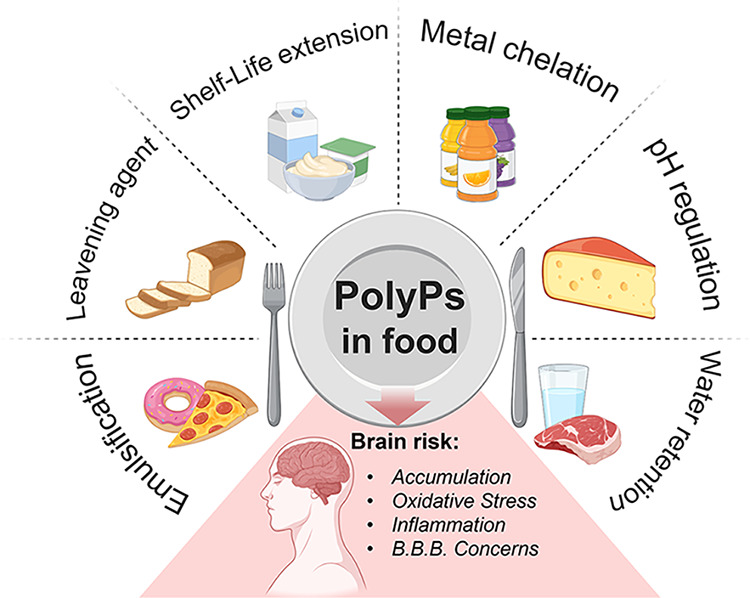
Representation of the diverse applications of PolyPs in the food industry and their potential implications for brain health.

Due to their physicochemical properties, polyPs can enhance the shelf life and sensory qualities of processed food.^18,21^ One of the primary uses of polyPs is to improve food moisture and texture by enhancing the water-holding capacity of proteins.^141,142^ For example, in meat products, they interact with myofibrillar proteins, promoting partial unfolding that exposes hydrophilic regions more inclined to interact with water, improving juiciness and texture.^142^ This property is also central to another role of polyPs, which is emulsification.^20,142^ PolyPs reduce surface tension between immiscible phases, such as fat and water, facilitating the formation and stabilization of emulsions, a critical feature in the production of homogenized sauces, dressings, and dairy products.^19–21,141,142^ Additionally, polyPs exhibit strong metal ion chelating abilities. Selectively binding undesirable cations (Ca^2+^, Mg^2+^, Fe^2+^, Cu^2+^) can prevent turbidity and sedimentation in beverages, and also oxidation of lipids in fatty foods, thus extending shelf life.^143–145^ Moreover, polyPs serve as pH regulators.^146,147^ They can control environmental acidity to inhibit the growth of pathogenic microorganisms, especially in meat and cheese products.^144,146,147^ Controlling acidity is also important for baked goods.^148–150^ In fact, polyPs facilitate the release of carbon dioxide from sodium bicarbonate, thus contributing to the rise and improved crumb structure of bread and pastries.^149,150^ Due to these diverse properties, polyPs are ubiquitous in modern food processing and are commonly found in multiple products, including processed meats, canned seafood, cheeses, carbonated drinks, and baked items.^21,146,147,150^

## 6 Connection between Exogenous Polyphosphates and Tauopathies

The extensive use of polyPs in the food industry and the emerging studies on their role during the onset of tauopathies raise concerning questions. The connections between diet and tauopathies have already been reported.^151–156^ However, none of these studies delved into the possible link between exogenous polyPs and the pathologies. The central issues are mainly twofold: (i) can intact exogenous polyPs be retained in the human body and (ii) can exogenous polyPs penetrate the blood-brain barrier (BBB). In physiological conditions, consumption of polyPs is generally recognized as safe within regulated limits, with 80–90% of dietary polyPs absorbed as free orthophosphate, and the excess excreted mainly by the kidneys.^22,23^ Additionally, even if polyPs were retained as whole polymers, their physicochemical properties exclude passive diffusion across an intact BBB. However, it has been reported that polyPs can disrupt endothelial barrier integrity in multiple organs, including kidneys and brain.^157^ Additionally, another study reported that high circulatory phosphate levels can be associated with cerebral small-vessel diseases.^158^ Therefore, a targeted analysis of exogenous polyP behavior in kidneys and their BBB-permeability under physiological conditions should be conducted. Unfortunately, elderly people and pathological patients steer away from physiological conditions, often presenting with BBB dysfunction and increased permeability.^24^ Moreover, these categories can also exhibit impairment in renal excretion of dietary phosphate.^159,160^ Therefore, it becomes fundamental to examine exogenous polyP breakdown, intake, and biodistribution in elderly individuals and pathological patients, with a specific focus on the interaction of polyPs with tau protein and mitochondria.

## 7 Future Perspectives and Conclusions

The evidence presented in this review highlights a pressing and underexplored side of tauopathy research: the emerging polyP-tau-mitochondria axis. This interaction connects biochemical dysregulation with potential nutritional contributors to neurodegeneration. Despite the long-standing perception of polyphosphates as safe food additives, mounting evidence has prompted a re-evaluation of their safety. This data suggest a possibility for polyphosphates to accelerate tau fibrillation and disrupt mitochondrial homeostasis, alimenting the vicious cycle of self-reinforcing pathology. These effects may be particularly deleterious in aging individuals or those with compromised blood-brain barrier integrity, as polyphosphates could pass the barrier in such cases. This perspective is particularly opportune considering the present limitations of therapeutic strategies for tauopathies, especially AD, underscoring the efficacy of prevention as a means of reducing the disease burden. Elucidation of the interplay among nutritional exposures, polyP metabolism, and neuronal vulnerability may yield novel opportunities for intervention at the earliest and most modifiable stages of tauopathies. Despite significant advancements, the molecular determinants that govern fibril formation and strain heterogeneity remain poorly predictable, particularly in the presence of physiologically relevant polyanions such as polyPs. Additionally, the connection between polyPs and mitochondrial dysfunction must be investigated further. Therefore, a critical future direction must include elucidating the neurobiological impact of both endogenous and exogenous polyPs using a combination of biochemical, structural, and cellular approaches. Possible experimental designs should include: (i) quantification and localization of endogenous polyPs in pathological samples,^161,162^ (ii) analysis of tau aggregation and mitochondrial dysfunction after manipulation of endogenous polyPs,^163–166^ (iii) biodistribution and BBB-transport studies of exogenous polyPs,^22,167^ and (iv) analysis of tau aggregation and mitochondrial dysfunction after administration of exogenous polyPs.^12,13,168,169^ Translational models, including retinal neurons and iPSC-derived human cells, offer a promising platform for investigating early pathogenic processes and mapping the influence of specific polyP species on tau conformation and mitochondrial function. In this context, retinal tissue can be particularly valuable, enabling noninvasive examination of early tau accumulation through routine imaging like optical coherence tomography.^170^ In fact, the retina shares the same embryonic origin as the central nervous system, therefore retinal tauopathy reflects brain alterations such as amyloid-β accumulation and tau hyperphosphorylation.^171^ The iPSC technology will create a humanized platform of retinal neurons to probe the polyP influence on tau aggregation while allowing real-time monitoring of polyP-induced tau conformational shifts. This approach can effectively translate preclinical insights into human tauopathy pathways.^172^ Another central challenge will be to define the molecular signatures underlying fibril polymorphism and identify the features that distinguish pathological strains from physiological aggregates. A considerable body of research has arrived at the conclusion that the propensity for tau aggregation may be partially encoded in the monomer structure. Consequently, future research should concentrate on integrating site-specific mutagenesis with molecular dynamics simulation supported by advanced structural biophysical methodologies.^173–175^ This knowledge will be crucial for the rational design of diagnostic probes and therapeutic agents as well as for improving the early detection of AD and enabling the development of scalable, cost-effective disease prevention strategies.
